# (*Z*)-3-(2-Amino­anilino)-1-phenyl­but-2-en-1-one

**DOI:** 10.1107/S160053681301060X

**Published:** 2013-04-24

**Authors:** Subramani Karthikeyan, Thothadri Srinivasan, Elumalai Sundaravadivel, Muthusamy Kandaswamy, Devadasan Velmurugan

**Affiliations:** a Centre of Advanced Study in Crystallography and Biophysics, University of Madras, Guindy Campus, Chennai 600 025, India; b Department of Inorganic Chemistry, University of Madras, Guindy Campus, Chennai 600 025, India

## Abstract

In the title compound, C_16_H_16_N_2_O, the phenyl and 2-amino­phenyl rings are almost perpendicular to one another, with a dihedral angle of 82.77 (8)°. There is an intra­molecular N—H⋯O hydrogen bond in the mol­ecule. In the crystal, mol­ecules are linked *via* N—H⋯O hydrogen bonds forming chains along [001]. There are also C—H⋯π inter­actions present, linking the chains to form a three-dimensional structure.

## Related literature

For the biological activity of chalcones, see: Di Carlo *et al.* (1999 ▶); Lin *et al.* (2002 ▶). For a related chalcone structure, see: Ranjith *et al.* (2010 ▶).
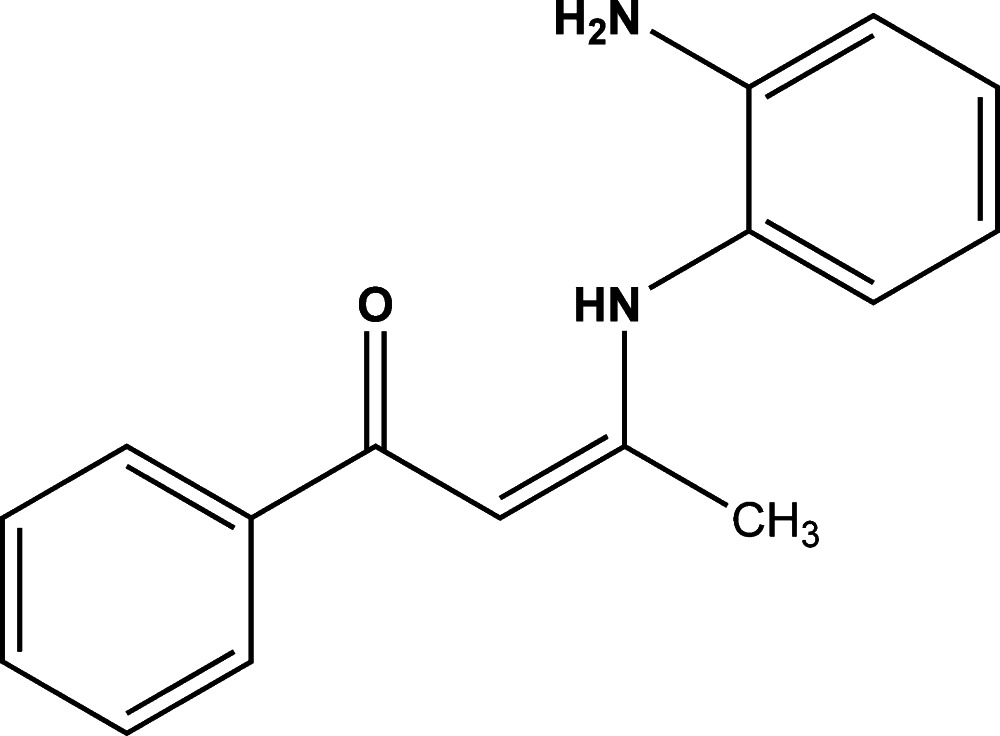


## Experimental

### 

#### Crystal data

C_16_H_16_N_2_O
*M* *_r_* = 252.31
Monoclinic, 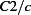

*a* = 15.489 (5) Å
*b* = 16.422 (5) Å
*c* = 11.684 (5) Å
β = 110.646 (5)°
*V* = 2781.1 (17) Å^3^
*Z* = 8
Mo *K*α radiation
μ = 0.08 mm^−1^
*T* = 293 K
0.30 × 0.25 × 0.20 mm

#### Data collection

Bruker SMART APEXII area-detector diffractometer
Absorption correction: multi-scan (*SADABS*; Bruker, 2008 ▶) *T*_min_ = 0.977, *T*_max_ = 0.985
18172 measured reflections
4023 independent reflections
2805 reflections with *I* > 2σ(*I*)
*R*_int_ = 0.028

#### Refinement

*R*[*F* ^2^ > 2σ(*F* ^2^)] = 0.050
*wR*(*F* ^2^) = 0.152
*S* = 1.01
4023 reflections
173 parameters
H-atom parameters constrained
Δρ_max_ = 0.30 e Å^−3^
Δρ_min_ = −0.22 e Å^−3^

### 

Data collection: *APEX2* (Bruker, 2008 ▶); cell refinement: *SAINT* (Bruker, 2008 ▶); data reduction: *SAINT*; program(s) used to solve structure: *SHELXS97* (Sheldrick, 2008 ▶); program(s) used to refine structure: *SHELXL97* (Sheldrick, 2008 ▶); molecular graphics: *ORTEP-3 for Windows* (Farrugia, 2012 ▶); software used to prepare material for publication: *SHELXL97* and *PLATON* (Spek, 2009 ▶).

## Supplementary Material

Crystal structure: contains datablock(s) global, I. DOI: 10.1107/S160053681301060X/su2583sup1.cif

Structure factors: contains datablock(s) I. DOI: 10.1107/S160053681301060X/su2583Isup2.hkl

Supplementary material file. DOI: 10.1107/S160053681301060X/su2583Isup3.cml

Additional supplementary materials:  crystallographic information; 3D view; checkCIF report

## Figures and Tables

**Table 1:** Hydrogen-bond geometry (Å, °) *Cg*1 is the centroid of ring C1–C6.

*D*—H⋯*A*	*D*—H	H⋯*A*	*D*⋯*A*	*D*—H⋯*A*
N2—H2*A*⋯O1	0.86	1.95	2.6311 (19)	135
N1—H1*A*⋯O1^i^	0.86	2.28	3.001 (2)	142
N1—H1*B*⋯O1^ii^	0.86	2.18	3.034 (2)	174
C14—H14⋯*Cg*1^iii^	0.93	2.96	3.773 (3)	147

Symmetry codes: (i) 
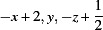
; (ii) 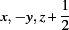; (iii) 
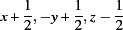
.

**Fig. 1.**
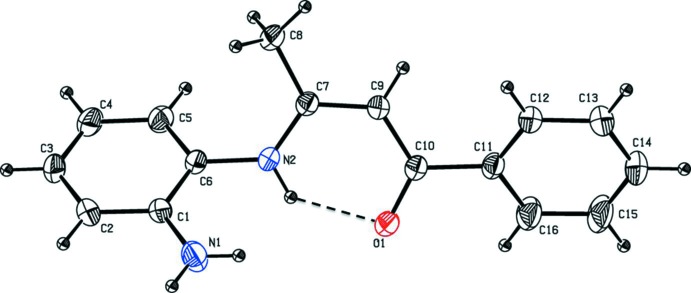
The molecular structure of the title molecule, with atom labelling. The displacement ellipsoids are drawn at the 30% probability level. The intramolecular N-H···O hydrogen bond is shown as a dashed line (see Table 1 for details).

**Fig. 2.**
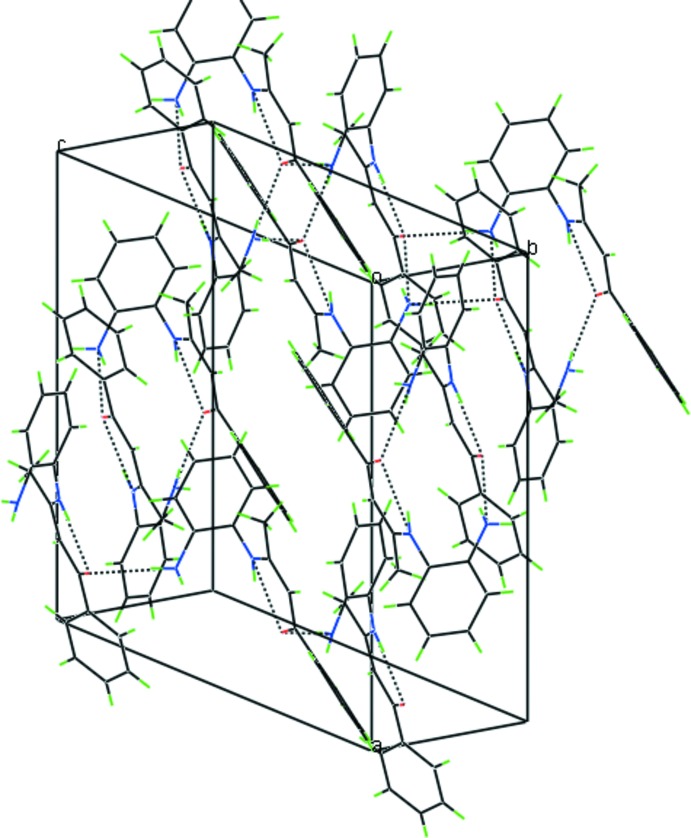
A view of the crystal packing of the title compound, showing the N-H···O hydrogen bonds as dashed lines (see Table 1 for details).

## supplementary crystallographic information

### Comment

Chalcones are a major class of natural products with widespread distribution in fruits, vegetables, spices, tea and soy based foodstuff and have recently been the subject of great interest for their interesting pharmacological activities (Di Carlo *et al.*, 1999). Chalcones and flavonoids have been reported to be anti-tuberculosis agents (Lin *et al.*, 2002). Against this background and in order to obtain detailed information on molecular conformations in the solid state of such compounds, an X-ray study of the title compound was carried out.

In the title compound, Fig. 1, the phenyl ring (C1-C6) makes a dihedral angle of 82.77 (8)° with the 2-aminophenyl ring (C11-C16), which shows that they are almost orthogonal to each other. The amine attached with phenyl ring (C11-C16) deviates by 0.0827 (14) Å. There is an intramolecular N-H···O hydrogen bond in the molecule (Table 1 and Fig. 1)

In the crystal, molecules are linked via N–H···O hydrogen bonds forming chains along the c axis direction. There are also C-H···π interactions present linking the chains to form a three-dimensional structure (see Table 1 and Fig. 2).

### Experimental

To a solution of 1-benzoylacetone (2 g, 12.3 mmol) in chloroform (25 ml), 1,2-diaminobenzene (1.33 g, 12.3 mmol) in chloroform (25 ml) was added with stirring. The yellow coloured solid product was collected by filtration and washed with water to remove unreacted 1,2-diaminobenzene. The microcrystalline compound was recrystallized from hot chloroform giving yellow crystals of the title compound, suitable for X-ray diffraction analysis, on slow evaporation of the solvent [Yield: 51%; M.p. 382 K].

### Refinement

Hydrogen atoms were placed in calculated positions and refined as riding atoms: N-H = 0.86 Å, C—H = 0.93-0.96 Å, with *U*_iso_(H) = 1.5*U*_eq_(C-methyl) and = 1.2U_eq_(N,C) for other H atoms.

### Crystal data

**Table:**

C_16_H_16_N_2_O	*F*(000) = 1072
*M**_r_* = 252.31	*D*_x_ = 1.205 Mg m^−^^3^
Monoclinic, *C*2/*c*	Mo *K*α radiation, λ = 0.71073 Å
Hall symbol: -C 2yc	Cell parameters from 4023 reflections
*a* = 15.489 (5) Å	θ = 2.3–30.0°
*b* = 16.422 (5) Å	µ = 0.08 mm^−^^1^
*c* = 11.684 (5) Å	*T* = 293 K
β = 110.646 (5)°	Block, colourless
*V* = 2781.1 (17) Å^3^	0.30 × 0.25 × 0.20 mm
*Z* = 8	

### Data collection

**Table:**

Bruker SMART APEXII area-detector diffractometer	4023 independent reflections
Radiation source: fine-focus sealed tube	2805 reflections with *I* > 2σ(*I*)
Graphite monochromator	*R*_int_ = 0.028
ω and φ scans	θ_max_ = 30.0°, θ_min_ = 2.3°
Absorption correction: multi-scan (*SADABS*; Bruker, 2008)	*h* = −21→21
*T*_min_ = 0.977, *T*_max_ = 0.985	*k* = −23→21
18172 measured reflections	*l* = −16→16

### Refinement

**Table:**

Refinement on *F*^2^	0 restraints
Least-squares matrix: full	Primary atom site location: structure-invariant direct methods
*R*[*F*^2^ > 2σ(*F*^2^)] = 0.050	Secondary atom site location: difference Fourier map
*wR*(*F*^2^) = 0.152	Hydrogen site location: inferred from neighbouring sites
*S* = 1.01	H-atom parameters constrained
4023 reflections	*w* = 1/[σ^2^(*F*_o_^2^) + (0.0752*P*)^2^ + 0.9814*P*] where *P* = (*F*_o_^2^ + 2*F*_c_^2^)/3
173 parameters	(Δ/σ)_max_ = 0.001

### Special details

**Table:**

Geometry. Bond distances, angles etc. have been calculated using the rounded fractional coordinates. All su's are estimated from the variances of the (full) variance-covariance matrix. The cell esds are taken into account in the estimation of distances, angles and torsion angles
Refinement. Refinement of F^2^ against ALL reflections. The weighted R-factor wR and goodness of fit S are based on F^2^, conventional R-factors R are based on F, with F set to zero for negative F^2^. The threshold expression of F^2^ > 2sigma(F^2^) is used only for calculating R-factors(gt) etc. and is not relevant to the choice of reflections for refinement. R-factors based on F^2^ are statistically about twice as large as those based on F, and R- factors based on ALL data will be even larger.

### Fractional atomic coordinates and isotropic or equivalent isotropic displacement parameters (Å^2^)

**Table:**

	*x*	*y*	*z*	*U*_iso_*/*U*_eq_	
O1	0.95043 (6)	0.11163 (6)	0.08924 (9)	0.0464 (3)	
N1	0.88576 (8)	0.00321 (8)	0.37211 (13)	0.0637 (5)	
N2	0.81921 (7)	0.11567 (7)	0.18340 (10)	0.0392 (3)	
C1	0.79279 (8)	0.01569 (8)	0.31971 (12)	0.0410 (4)	
C2	0.73017 (10)	−0.02773 (9)	0.35822 (14)	0.0490 (4)	
C3	0.63692 (10)	−0.01773 (9)	0.30190 (15)	0.0527 (5)	
C4	0.60208 (9)	0.03447 (10)	0.20489 (15)	0.0546 (5)	
C5	0.66227 (9)	0.07806 (9)	0.16509 (13)	0.0470 (4)	
C6	0.75672 (8)	0.07045 (8)	0.22326 (11)	0.0375 (3)	
C7	0.82154 (8)	0.19629 (8)	0.17067 (10)	0.0356 (3)	
C8	0.75857 (10)	0.24833 (9)	0.21128 (14)	0.0472 (4)	
C9	0.88265 (8)	0.23183 (8)	0.12356 (11)	0.0383 (4)	
C10	0.94545 (8)	0.18800 (8)	0.08424 (10)	0.0362 (3)	
C11	1.00769 (9)	0.23272 (8)	0.03291 (11)	0.0407 (4)	
C12	1.03522 (9)	0.31218 (9)	0.06381 (13)	0.0464 (4)	
C13	1.09525 (11)	0.35058 (10)	0.01689 (16)	0.0580 (5)	
C14	1.12735 (13)	0.30984 (12)	−0.06213 (17)	0.0672 (7)	
C15	1.10050 (14)	0.23138 (13)	−0.09387 (18)	0.0786 (8)	
C16	1.04137 (12)	0.19230 (11)	−0.04670 (15)	0.0624 (6)	
H1A	0.92310	0.02930	0.34570	0.0760*	
H1B	0.90690	−0.03080	0.43150	0.0760*	
H2	0.75230	−0.06400	0.42320	0.0590*	
H2A	0.85990	0.08800	0.16560	0.0470*	
H3	0.59670	−0.04670	0.32980	0.0630*	
H4	0.53870	0.04040	0.16640	0.0650*	
H5	0.63910	0.11290	0.09850	0.0560*	
H8A	0.69690	0.24440	0.15290	0.0710*	
H8B	0.77890	0.30390	0.21730	0.0710*	
H8C	0.75950	0.23010	0.28970	0.0710*	
H9	0.88240	0.28830	0.11740	0.0460*	
H12	1.01320	0.34040	0.11690	0.0560*	
H13	1.11370	0.40400	0.03910	0.0700*	
H14	1.16730	0.33550	−0.09410	0.0810*	
H15	1.12220	0.20390	−0.14790	0.0940*	
H16	1.02410	0.13860	−0.06850	0.0750*	

### Atomic displacement parameters (Å^2^)

**Table:**

	*U*^11^	*U*^22^	*U*^33^	*U*^12^	*U*^13^	*U*^23^
O1	0.0449 (5)	0.0420 (5)	0.0611 (6)	0.0004 (4)	0.0298 (4)	−0.0034 (4)
N1	0.0373 (6)	0.0634 (9)	0.0840 (10)	0.0005 (5)	0.0135 (6)	0.0327 (7)
N2	0.0351 (5)	0.0400 (6)	0.0495 (6)	0.0026 (4)	0.0237 (4)	0.0041 (4)
C1	0.0375 (6)	0.0356 (7)	0.0518 (7)	−0.0017 (5)	0.0182 (5)	0.0021 (5)
C2	0.0513 (8)	0.0400 (7)	0.0604 (8)	−0.0043 (6)	0.0255 (6)	0.0080 (6)
C3	0.0473 (7)	0.0468 (8)	0.0748 (10)	−0.0095 (6)	0.0348 (7)	0.0005 (7)
C4	0.0337 (6)	0.0576 (9)	0.0748 (10)	−0.0036 (6)	0.0221 (6)	−0.0007 (7)
C5	0.0369 (6)	0.0525 (8)	0.0519 (7)	0.0013 (6)	0.0160 (6)	0.0046 (6)
C6	0.0348 (6)	0.0377 (6)	0.0448 (6)	−0.0015 (5)	0.0201 (5)	−0.0002 (5)
C7	0.0333 (5)	0.0413 (7)	0.0335 (5)	0.0019 (5)	0.0133 (4)	0.0015 (5)
C8	0.0465 (7)	0.0465 (8)	0.0581 (8)	0.0027 (6)	0.0304 (6)	−0.0030 (6)
C9	0.0404 (6)	0.0371 (7)	0.0422 (6)	0.0013 (5)	0.0207 (5)	0.0033 (5)
C10	0.0337 (5)	0.0422 (7)	0.0343 (5)	−0.0008 (5)	0.0139 (4)	0.0005 (5)
C11	0.0378 (6)	0.0504 (8)	0.0382 (6)	0.0014 (5)	0.0186 (5)	0.0048 (5)
C12	0.0467 (7)	0.0474 (8)	0.0512 (7)	0.0013 (6)	0.0250 (6)	0.0055 (6)
C13	0.0572 (9)	0.0535 (9)	0.0714 (10)	−0.0037 (7)	0.0326 (8)	0.0115 (7)
C14	0.0705 (11)	0.0736 (12)	0.0761 (11)	−0.0036 (9)	0.0490 (9)	0.0162 (9)
C15	0.0961 (14)	0.0872 (14)	0.0837 (12)	−0.0094 (11)	0.0705 (12)	−0.0065 (10)
C16	0.0758 (11)	0.0638 (10)	0.0667 (10)	−0.0100 (8)	0.0487 (9)	−0.0088 (8)

### Geometric parameters (Å, º)

**Table:**

O1—C10	1.2566 (17)	C11—C12	1.381 (2)
N1—C1	1.367 (2)	C12—C13	1.386 (2)
N2—C6	1.4224 (18)	C13—C14	1.368 (3)
N2—C7	1.3342 (18)	C14—C15	1.365 (3)
N1—H1A	0.8600	C15—C16	1.382 (3)
N1—H1B	0.8600	C2—H2	0.9300
N2—H2A	0.8600	C3—H3	0.9300
C1—C2	1.400 (2)	C4—H4	0.9300
C1—C6	1.3953 (19)	C5—H5	0.9300
C2—C3	1.370 (2)	C8—H8A	0.9600
C3—C4	1.371 (2)	C8—H8B	0.9600
C4—C5	1.380 (2)	C8—H8C	0.9600
C5—C6	1.384 (2)	C9—H9	0.9300
C7—C8	1.494 (2)	C12—H12	0.9300
C7—C9	1.3809 (19)	C13—H13	0.9300
C9—C10	1.4108 (19)	C14—H14	0.9300
C10—C11	1.495 (2)	C15—H15	0.9300
C11—C16	1.386 (2)	C16—H16	0.9300
			
C6—N2—C7	127.08 (12)	C14—C15—C16	120.76 (19)
C1—N1—H1A	120.00	C11—C16—C15	120.30 (17)
C1—N1—H1B	120.00	C1—C2—H2	119.00
H1A—N1—H1B	120.00	C3—C2—H2	119.00
C7—N2—H2A	117.00	C2—C3—H3	120.00
C6—N2—H2A	116.00	C4—C3—H3	120.00
N1—C1—C6	121.13 (12)	C3—C4—H4	120.00
C2—C1—C6	117.54 (13)	C5—C4—H4	120.00
N1—C1—C2	121.30 (13)	C4—C5—H5	120.00
C1—C2—C3	121.15 (14)	C6—C5—H5	120.00
C2—C3—C4	120.88 (15)	C7—C8—H8A	109.00
C3—C4—C5	119.16 (14)	C7—C8—H8B	109.00
C4—C5—C6	120.69 (13)	C7—C8—H8C	109.00
N2—C6—C5	121.03 (12)	H8A—C8—H8B	110.00
C1—C6—C5	120.50 (12)	H8A—C8—H8C	109.00
N2—C6—C1	118.43 (12)	H8B—C8—H8C	109.00
N2—C7—C8	119.06 (12)	C7—C9—H9	118.00
N2—C7—C9	120.87 (12)	C10—C9—H9	118.00
C8—C7—C9	120.05 (12)	C11—C12—H12	120.00
C7—C9—C10	124.22 (12)	C13—C12—H12	119.00
C9—C10—C11	119.74 (12)	C12—C13—H13	120.00
O1—C10—C11	118.11 (12)	C14—C13—H13	120.00
O1—C10—C9	122.15 (12)	C13—C14—H14	120.00
C10—C11—C12	122.86 (12)	C15—C14—H14	120.00
C12—C11—C16	118.27 (14)	C14—C15—H15	120.00
C10—C11—C16	118.85 (13)	C16—C15—H15	120.00
C11—C12—C13	120.99 (14)	C11—C16—H16	120.00
C12—C13—C14	119.90 (16)	C15—C16—H16	120.00
C13—C14—C15	119.79 (19)		
			
C6—N2—C7—C9	−175.89 (12)	C8—C7—C9—C10	179.21 (12)
C7—N2—C6—C1	−127.15 (14)	C7—C9—C10—O1	0.06 (19)
C7—N2—C6—C5	55.26 (18)	C7—C9—C10—C11	179.11 (11)
C6—N2—C7—C8	5.71 (19)	O1—C10—C11—C12	−154.23 (13)
C6—C1—C2—C3	1.0 (2)	O1—C10—C11—C16	24.03 (18)
N1—C1—C6—N2	−2.42 (19)	C9—C10—C11—C12	26.69 (18)
N1—C1—C6—C5	175.19 (13)	C9—C10—C11—C16	−155.06 (13)
N1—C1—C2—C3	−177.06 (14)	C10—C11—C12—C13	178.04 (14)
C2—C1—C6—N2	179.49 (12)	C16—C11—C12—C13	−0.2 (2)
C2—C1—C6—C5	−2.9 (2)	C10—C11—C16—C15	−178.72 (15)
C1—C2—C3—C4	0.8 (2)	C12—C11—C16—C15	−0.4 (2)
C2—C3—C4—C5	−0.9 (2)	C11—C12—C13—C14	0.6 (2)
C3—C4—C5—C6	−1.1 (2)	C12—C13—C14—C15	−0.4 (3)
C4—C5—C6—N2	−179.49 (13)	C13—C14—C15—C16	−0.3 (3)
C4—C5—C6—C1	3.0 (2)	C14—C15—C16—C11	0.6 (3)
N2—C7—C9—C10	0.83 (19)		

### Hydrogen-bond geometry (Å, º)

Cg1 is the centroid of ring C1–C6.

**Table:**

*D*—H···*A*	*D*—H	H···*A*	*D*···*A*	*D*—H···*A*
N2—H2*A*···O1	0.86	1.95	2.6311 (19)	135
N1—H1*A*···O1^i^	0.86	2.28	3.001 (2)	142
N1—H1*B*···O1^ii^	0.86	2.18	3.034 (2)	174
C14—H14···*Cg*1^iii^	0.93	2.96	3.773 (3)	147

Symmetry codes: (i) −*x*+2, *y*, −*z*+1/2; (ii) *x*, −*y*, *z*+1/2; (iii) *x*+1/2, −*y*+1/2, *z*−1/2.
